# *Heteropterys cotinifolia*: A Neuropharmacological and Phytochemical Approach with Possible Taxonomic Implications

**DOI:** 10.1155/2013/870468

**Published:** 2013-12-19

**Authors:** Maira Huerta-Reyes, Alejandro Zamilpa, Rafael Álvarez-Chimal, José Ángel Luna-Manzanares, María Esther León-Velasco, Arturo Aguilar-Rojas, Manuel Jiménez-Estrada, María Guadalupe Campos-Lara

**Affiliations:** ^1^Centro de Investigación Biomédica del Sur, Instituto Mexicano del Seguro Social, Argentina No. 1, Col. Centro, CP 62790 Xochitepec, MOR, Mexico; ^2^Unidad de Investigación Médica en Farmacología, Hospital de Especialidades, Centro Médico Nacional Siglo XXI, Instituto Mexicano del Seguro Social, Avenida Cuauhtémoc No. 330, Col. Doctores, Del. Cuauhtémoc, CP 06720, DF, Mexico; ^3^Instituto de Química, Universidad Nacional Autónoma de México, Del. Coyoacán 04510, DF, Mexico; ^4^Universidad Tominaga Nakamoto, Luis Freg No. 6 y 12, Col. Lomas de Sotelo, CP 53390, Naucalpan de Juárez, Estado de México, Mexico; ^5^Herbario Nacional de México (MEXU), Instituto de Biología, Universidad Nacional Autónoma de México, Apartado Postal 70-367, Del. Coyoacán 04510, DF, Mexico; ^6^Unidad de Investigación Médica en Medicina Reproductiva, UMAE en Ginecología y Obstetricia No. 4, Instituto Mexicano del Seguro Social, Avenida Río Magdalena No. 289, Col. Tizapán San Ángel, CP 01090, Delegación Álvaro Obregón, DF, Mexico

## Abstract

*Heteropterys cotinifolia* (Malpighiaceae) has been used in traditional Mexican medicine mainly for the treatment of nervous disorders. However, the specific neuropharmacological activities responsible for this use remain to be defined. The present study evaluates the antidepressant and anxiolytic effects produced by the methanolic extract of *Heteropterys cotinifolia* and the influence of such effects on motor activity in ICR mice. Our results show that the methanolic extract of *Heteropterys cotinifolia* produces a dose-dependent antidepressant effect in the forced swimming test in mice at doses from 31 to 310 mg/kg, with no reduction of mice locomotion. However, no anxiolytic properties were observed. Our findings suggest that the main extract compounds identified as chlorogenic acid and rutin may be involved in the antidepressant effects. To our knowledge, the present study constitutes the first report of pharmacological and phytochemical data of *Heteropterys cotinifolia*. The presence of flavonoids in the methanolic extract of *Heteropterys cotinifolia* may also provide further data to characterize taxonomically this species in order to be distinguished from others species closely related and belonging to the same genus.

## 1. Introduction

Depression and anxiety are the most frequent mental disorders in all communities all over the world. Around 350 million people are estimated to suffer from depression [1], while anxiety affects one eight of the population worldwide [2]. The prevalence of comorbidity of depression and anxiety is high and by far the most frequent mixture of symptoms among mental disorders [3]. The current pharmacological treatments for depression and anxiety are effective only in a certain percentage of the population and, moreover, most drugs have many adverse side effects including hypotension, arrhythmias, insomnia and sexual dysfunction in the case of antidepressant synthetic nitrogen-bearing compounds [4], and sedation, muscle relaxation, amnesia, and physical dependence when benzodiazepines are used [5]. Therefore, there is a need for research and development of more effective pharmacotherapies with little or no side effects.

*Heteropterys cotinifolia* A. Juss is a woody vine belonging to the Malpighiaceae family and has been used in traditional Mexican medicine mainly for the treatment of nervous disorders [6]. Since the taxonomic point of view, *Heteropterys cotinifolia* presents considerable variability which probably explains why it has been described many times [7]. However, recent geographical data seem to contribute in the definition of *Heteropterys cotinifolia* as an endemic species of Mexico [8]. To the best of our knowledge, there is no information about scientific evidence that shows the possible biological properties of *Heteropterys cotinifolia. *Thus, the present study focuses on the neuropharmacological activities of *Heteropterys cotinifolia* to understand its traditional medicinal applications. The study was conducted to evaluate the antidepressant and anxiolytic effects, as well as the influence on the motor activity produced by the extract of *Heteropterys cotinifolia* in ICR mice using a variety of models, such as the elevated plus maze, the forced swimming test, and the open field test. The extract of *Heteropterys cotinifolia* was also analyzed by HPLC to quantify its main constituents.

## 2. Materials and Methods

### 2.1. Plant Collection and Identification

The aerial parts of *Heteropterys cotinifolia *A. Juss. were collected from the state of Morelos, Mexico, in October 2007. The identification of the plant was authenticated by an expert in the field of plant taxonomy, who is also one of the authors (M.E. León-Velasco). A voucher was deposited as reference at the Mexican Institute of Social Security Medicinal Herbarium (IMSSM) with the number 15451.

### 2.2. Preparation of Extracts

The plant material was dried in darkness and at room temperature and subsequently powered (3000 g). After dewaxing with *n*-hexane, the plant material was successively extracted (3x) overnight with methanol (100%). The extraction volume used was 7.5 L of solvent per each kg of plant material. The liquid extract was dried by removal of the solvent under vacuum. The methanolic extract of *Heteropterys cotinifolia* (HcMeOH) (257.7 g) was then used in the pharmacological experiments.

### 2.3. Animals

The animal experiments were performed in strict adherence to the official requirements of the Mexican Regulations of Experimental Animal Care (NOM-062-ZOO-1999). The experimental protocol was approved by the institutional research and ethics committees (Registry number 2010-1701-60). For each neuropharmacological assay, groups of ten ICR albino mice weighing 20–30 g each were housed in community cages and maintained under regular laboratory conditions (22 ± 1°C, 12 h light-dark cycle, free access to water and standard rodent chow). All animals were acclimatized over 3 weeks prior to initiation of the test. The experiments were conducted in a special adjacent noise-free room with controlled illumination. Each animal was used only once in the experiment.

### 2.4. Neuropharmacological Assays

HcMeOH was administrated *p.o.* at a dose of 310 mg/kg. In those assays exhibiting activity at the initial dose of 310 mg/kg, the dose-dependent effects were determined by using 100 and 31 mg/kg doses. The control substances were diazepam (DZP), imipramine hydrochloride (IMI), and saline solution (ss) as vehicle. HcMeOH and control substances were dissolved in ss.

#### 2.4.1. Forced Swimming Test (FST)

Among all animal models, the FST remains one of the most used tools for screening antidepressants [9, 10]. The apparatus utilized to perform the FST consisted of a clear glass cylinder (30 cm high × 12 cm diameter) with water filled to a depth of 15 cm (25 ± 3°C). The mice were treated with HcMeOH (31, 100 and 310 mg/kg, experimental treatment, *n* = 10) and ss 0.1% (vehicle, *p*.*o.*, control group, *n* = 10) 1 h prior to the test. IMI (15 mg/kg, *i.p*., positive control, *n* = 10) was administered 30 min before the test. During the test session, a trained observer recorded the immobility time.

#### 2.4.2. Elevated Plus Maze (EPM)

The EPM test is one of the most commonly used animal models of anxiety-like behavior [11]. The maze was constructed of Plexiglas and consisted of a central platform (5 × 5 cm) with two open (30 × 5 cm) and two closed arms (30 × 5 cm) and 25 cm high walls. The maze was elevated 38.5 cm from the floor. The mice were treated 30 min prior to the test with DZP (1 mg/kg, *i.p*., positive control, *n* = 10), while ss 0.1% (vehicle, *p.o.*, control group, *n* = 10) and HcMeOH (310 mg/kg, experimental treatment, *n* = 10) were administrated 1 h prior to the test. Each animal was placed at the center of the maze facing one of the open arms. The number of entries and the time spent in the enclosed and open arms were recorded during the 5 min test.

#### 2.4.3. Open Field Test (OFT)

The open field area was comprised of an opaque-Plexiglas box (60 × 60 × 35 cm) divided into nine squares of equal size. In this test, the HcMeOH was administered at doses of 310, 100 and 31 mg/kg (experimental treatment, *n* = 10), DZP at 1 mg/kg (positive control, *n* = 10) and ss 0.1% (control group, *n* = 10), 60 min before the beginning of the test [12]. The open field test was used to evaluate the locomotor activity of mice that had previously been subjected to the FST and EPM tests.

#### 2.4.4. Statistical Analysis

All data were represented as mean ± standard deviation (S.D.). Data were analyzed by one-way ANOVA followed by Dunnett's test for comparisons against control. Values of *P*≤ 0.05 (∗) were considered statistically significant.

### 2.5. HPLC Analysis

We performed an HPLC analysis of HcMeOH for detection and quantification of its major constituents. HPLC analysis was conducted on a Waters 2695 liquid chromatographer equipped with a Waters 2996 photodiode array detector. Separation was carried out by using a RP C-18 Superspher (Merck) column (240 × 4 mm; 5 *μ*m) with the following solvent ratios for the mobile phase, where solvent A is 0.1% acetic acid and solvent B is acetonitrile: A : B = 100 : 0 (0–3 min); 90 : 10 (4-5 min); 80 : 20 (6–9 min); 0 : 100 (10–12 min); and 100 : 0 (13–15 min). The sample injection volume was 10 *μ*L with a 1.0 mL/min flow rate. The peak analysis and assignment were performed using commercial standard compounds, which were identified in accordance with their UV spectra and retention time (*t*_*R*_) in the HPLC chromatogram. The detection wavelength was scanned at 190–400 nm. Quantification of the main compounds was achieved by calibrating curves that were separately constructed with pure standards.

## 3. Results and Discussion

**Figure 1:**
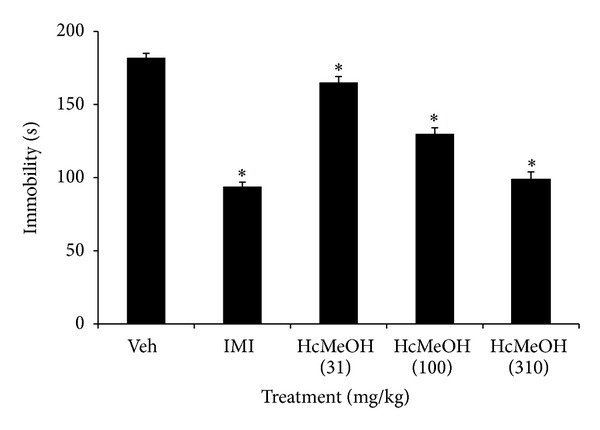
Effect of oral administration of *Heteropterys cotinifolia* methanolic extract on immobility time of ICR mice exposed to FST. *P < 0.05 with ANOVA followed by a *post hoc* Dunnett test (mean ± S.D.). Veh, vehicle; IMI, imipramine hydrochloride; HcMeOH, methanolic extract of *Heteropterys cotinifolia. *

**Figure 2:**
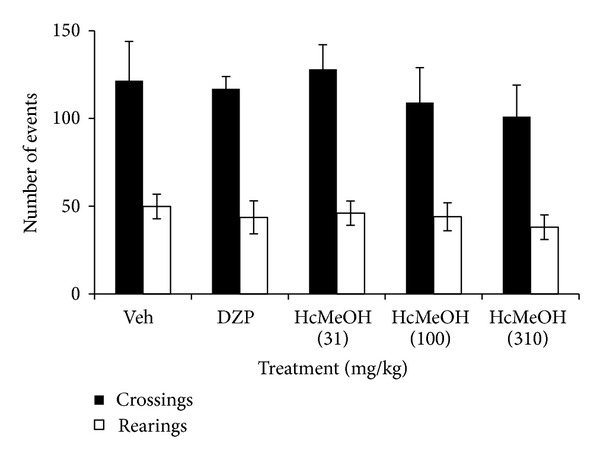
Effect of oral administration of *Heteropterys cotinifolia* methanolic extract on the number of total crossings and rearings of ICR mice exposed to OFT. *P < 0.05 with ANOVA followed by a *post hoc* Dunnett test (mean ± S.D.). Veh, vehicle; DZP, diazepam; HcMeOH, methanolic extract of *Heteropterys cotinifolia. *

The present study investigated for the first time the CNS effects of the methanolic extract of the aerial parts of *Heteropterys cotinifolia* (HcMeOH) in mice. The FST is the most widely used and recognized pharmacological model, for assessing antidepressant activities. The development of immobility when mice were placed inside an inescapable cylinder filled with water reflects the cessation of persistent escape-directed behavior [9, 13]. Our results showed that HcMeOH induced a significant antidepressant effect in a dose-dependent manner in the FST since it significantly diminished the immobility time compared with the control group (Veh) (*P* < 0.05) at doses from 31 to 310 mg/kg (Figure 1). It is noteworthy that in the FST test, false positive results can be obtained for agents that stimulate locomotor activity [14]. Therefore, the observation that HcMeOH did not increase the number of crossings and rearings in the OFT (Figure 2) confirms the assumption that the antidepressant effect of the extract in the FST is specific [15].

**Figure 3:**
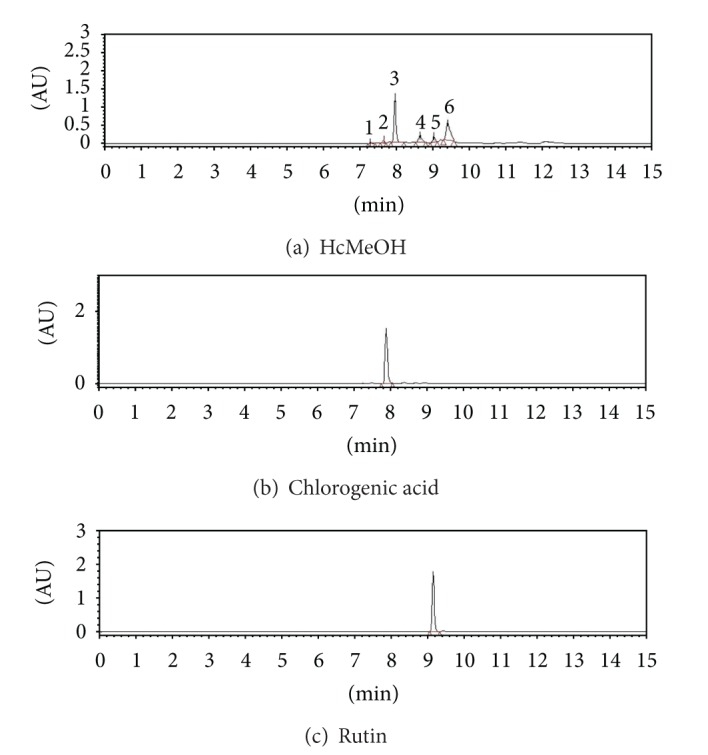
HPLC chromatogram comparison between HcMeOH and commercial standards. (a) Crude extract of HcMeOH; (b) chlorogenic acid commercial standard; (c) rutin commercial standard.

The HPCL analysis showed two main compounds in the active extract HcMeOH (Figure 3(a)). The first (peak 3) was identified and quantified as chlorogenic acid (36.4 mg/g) (Figure 3(b)) while the second main compound (peak 6) was recognized as the flavonoid rutin (17.9 mg/g) (Figure 3(c)), since the UV spectra and *t*_*R*_ values were in agreement with the commercial pure standards. Quantification was established with calibration curves (linear regression where *r* ^2^ > 0.9932). The identity of the rest of the compounds (peaks 1, 2, 4, and 5) present in HcMeOH (Figure 3(a)) remains unknown as its *t*_*R*_ and UV absorption values did not match with any of the commercial standards used additionally. Likewise, the scarce amounts observed, particularly for peaks 1 and 2, made difficult the identification (Table 1). Although we cannot rule out the participation of other components in the antidepressant effect of HcMeOH, our results suggest a significant role for chlorogenic acid and rutin in the antidepressant properties of HcMeOH due to its presence as major constituents. Rutin has been shown to play an essential role in the antidepressant activity of plant extracts widely recognized as antidepressants, such as *Hypericum perforatum*, participating in the enhancement of the bioavailability of other compounds present in the extract to confer the entire biological activity in the FST [16]. The involvement of the serotonergic, noradrenergic, and/or dopaminergic systems in the synaptic cleft has been reported as the mechanism responsible of the antidepressant actions of rutin [17]. Similarly, the potent antidepressant effect of chlorogenic acid, the other major compound detected in HcMeOH, has been exhibited in FST when orally administered [18]. Therefore, it can be suggested that the antidepressant effect observed in the present work may largely be due to the chlorogenic acid and/or rutin content detected in HcMeOH. Our findings are in accordance with reports on chlorogenic acid and rutin as the main components of extracts widely recognized as antidepressants such as *Hypericum *extracts [19–21]. Moreover, in the case of *Hypericum origanifolium*, these two components are also the main compounds found in the crude extract, even over hypericin and hyperforin, the compounds described by far as the responsible for the antidepressant properties of *Hypericum perforatum* (St. John's wort) [22].

On the other hand, the findings of the present study contribute to place the species belonging to the *Heteropterys* genus as a potential source of extracts able to relieve the affections of CNS. For example, the Brazilian species, *Heteropterys aphrodisiaca* O. Mach. improved learning and memory deficits in aged rats [23], and has also showed a strong reduction in the oxidative stress in young and old rat brains [24], whereas the species *Heteropterys glabra* Hook & Arn. induced a reduction in motor activity and alterations in EEG parameters [25]. Recently, our work group described the antidepressant, anxiolytic, and anticonvulsant activities in mice without toxicity effects of the methanolic extract of *Heteropterys brachiata* (L.) DC. [26]. However, the hydroalcoholic extracts of roots, branches, and leaves of *Heteropterys tomentosa* A. Juss showed no effects when evaluated in animal models of stress and learning/memory [27], and no influence on the apoptosis of the hippocampal cells of aged rats [28]. Interestingly, even when *Heteropterys *species could be considered relevant from the neuropharmacological perspective, the studies about the chemical composition of the active CNS extracts are scarce. Partial phytochemical screenings for detecting chemical groups such as glycosides, polyphenols, tannins and alkaloids saponins, and anthracene derivatives in *Heteropterys aphrodisiaca* have been reported [23]. The hydroxycinnamic acids, chlorogenic acid, and chlorogenic acid methyl ester as responsible for the neuropharmacological properties in *Heteropterys brachiata *have been described [26]. However, although chlorogenic acid is the major compound identified in HcMeOH in the present work, it is likely that the differences observed in neuroactivity compared with those elicited by the methanolic extract of *Heteropterys brachiata* may be due to the presence of flavonoids that appear to be the second main compound group in HcMeOH; that is, the synergistic effects are crucial for the observation of neuropharmacological activity as has been reported widely elsewhere [16].

**Figure 4:**
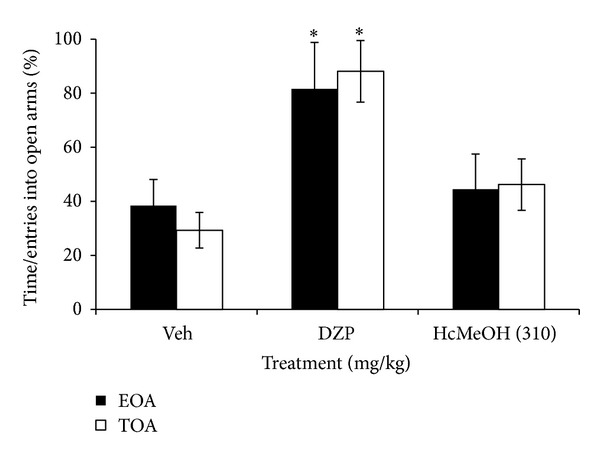
Effect of oral administration of *Heteropterys cotinifolia *methanolic extraction the percentage of time spent in open arms (TOA) and entries into open arms (EOA) by mice exposed to EPM. *P < 0.05 in ANOVA followed by a *post hoc* Dunnett test (mean ± S.D.). Veh, vehicle; DZP, diazepam; HcMeOH, methanolic extract of *Heteropterys cotinifolia*.

Regarding the third test, our results show that HcMeOH does not elicit anxiolytic effects at the dose tested in EPM since no changes were detected in the percentage of Time that mice spent in Open Arms (TOA) and the percentage of Entries into the Open Arms (EOA) with respect to the control group (Veh) (*P* > 0.05) (Figure 4).

In addition to the neuropharmacological relevance of the *Heteropterys* extracts, the chemical profiles have been useful in the definition of the species belonging to this genus from the taxonomic point of view [29]. Thus, the chemical composition consisting of chlorogenic acid and rutin as main compounds of HcMeOH may provide additional data to characterize the species to be distinguished from the closely related *Heteropterys brachiata* in which, in contrast, flavonoids were not detected in the methanolic extract. Consequently, our results may also contribute to provide elements for a quick and simple identification of *Heteropterys cotinifolia *species even in the field collecting zone, where pink flowers and fruits, frequently cause confusion with *Heteropterys brachiata*. Then, at collecting time and in combination with the morphological characters, a positive detection of flavonoids by simple TLC could be useful to distinguish *Heteropterys cotinifolia *from other close species. This discernment technique by TLC has been used successfully in other plant species with relevant biological properties [30].

## 4. Conclusions

The methanolic extract of *Heteropterys cotinifolia* possesses antidepressant properties in which chlorogenic acid and rutin could be involved. To the best of our knowledge, this is the first report of the biological activities and chemical data of this species. Our findings support the pharmacological justification for the traditional use of *Heteropterys cotinifolia* in the treatment of nervous disorders and encourage further studies for the development of this extract as a therapeutic agent.

## Figures and Tables

**Table 1:** Retention times and absorbance values of peaks detected in HcMeOH.

Peak	Retention time (min)*	*λ* (nm)
1	7.29	(213, 220, 273)
2	7.65	(218, 240, 325)
3	7.96	(218, 242, 325)
4	8.65	(218, 242, 325)
5	9.03	(220, 243, 329)
6	9.41	(210, 255, 355)
Chlorogenic acid (commercial pure standard)	7.88	(218, 242, 325)
Rutin (commercial pure standard)	9.14	(210, 255, 355)

*Retention time window <5% was employed to identify each compound.
